# Health status of senior netball players, their medication use and attitudes towards doping

**DOI:** 10.3389/fspor.2024.1436080

**Published:** 2024-09-03

**Authors:** Micaela de Abreu, Kim Nolte, Dina Christa Janse van Rensburg, Xan Swart

**Affiliations:** ^1^ Department of Physiology (Division of Biokinetics and Sport Science), Faculty of Health Sciences, University of Pretoria, Pretoria, South Africa; ^2^ Section Sports Medicine, Faculty of Health Sciences, University of Pretoria, Pretoria, South Africa

**Keywords:** netball, doping, medication, chronic disease, doping attitudes

## Abstract

Limited research exists on the health and injuries of South African senior netball players. Senior netball players may be at greater risk of injuries and chronic disease due to their age. To treat these conditions, they may use prescription and over-the-counter (OTC) medications and, therefore, may be more vulnerable to unintentional doping. The primary aim of this study was to determine the health status, medication use and attitudes towards doping of South African senior netball players. A cross-sectional descriptive design was employed to collect data by means of an online survey. The validated 8-item Performance Enhancement Attitudes Scale (PEAS) was used to gather information on the netball player's attitudes towards doping. Descriptive statistics were used to describe the data using proportions (categorical), means (normally distributed, continuous) and medians (non-normal distributed, continuous). Doping prevalence and accompanying 95% confidence interval were calculated. Sixty senior netball players consented and completed the self-report questionnaire. The prevalence of chronic disease was 11.67%. Asthma and other conditions such as depression and attention-deficit/hyperactivity (ADHD) had the highest prevalence of 3.33%. The prevalence of chronic prescription medication use was 8.33% and 66.67% of the netball players reported receiving prescription injections, medications or utilizing OTC medications for treating injury or illness suffered 1–6 weeks before or during competition. The netball players do not have a lenient attitude towards doping. The prescription and OTC medication use could put this cohort of netball players at risk of unintentional doping. Anti-doping education aimed at senior athletes may be beneficial to reduce the risk of unintentional doping due to prescription and OTC medication use for injury or illness.

## 1 Introduction

Netball's physical demands are characterized as dynamic, high-intensity and intermittent (1, 2). Netball is a team-based sport that is played over 60 min in elite level ranks (1, 2). A team consists of 7 positions, namely: Goal Shooter, Goal Attack, Wing Attack, Centre, Wing Defense, Goal Defense, and Goal Keeper (1, 2). Together, the physical, technical and tactical demands of the sport impose unique mental skills and physicality from netball players (2).

Medication (prescription and OTC) and nutritional supplement use may assist athletes in maintaining optimal health and performance and assist in rapid recovery from injury and illness (3–5). Nutritional supplements may include vitamin, mineral, herbal powder, carbohydrate and protein powder preparations that do not contain anabolic agents prohibited by the World Anti-doping Agency (WADA) (6). Research indicates that the incidence of prescription medication use by athletes is 20% higher than that of the general population. Upon searching the literature, no studies on prescription and OTC medication use in netball players specifically could be found.

Athletes competing at collegiate and elite level ranks consume more nutritional supplements compared to sedentary or physically active populations. Netball players have reported taking nutritional supplements to maintain health, as part of a dietary routine, to boost immunity, from peer recommendations, to improve energy and performance, to reduce fatigue, to improve strength, for sponsorship endorsements and for travel requirements (7). Additionally, medication (prescription and OTC) and nutritional supplement use are common amongst athlete populations, as well as amongst the older general populations (8). Older athletes have also been reported to utilize diuretics, statins and beta-adrenergic blocking agents for the treatment and management of chronic diseases, and may make them vulnerable to committing unintentional doping violations (8).

The detection, usage and possession of prohibited performance enhancing drugs (PEDs) and methods to improve performance or attempt to influence doping test results are collectively termed “doping” and are banned by national and international sport governing bodies and by WADA (9–12). The percentage of athletes that test positive for doping remains consistent at 1%–2% annually (13–15).

No studies measuring attitudes towards doping, prescription and OTC medication and nutritional supplement use in netball could be sourced. Information on health status, prescription and OTC medication use and attitudes towards doping in South African senior netball players may guide future anti-doping programs and interventions in this cohort, as well as other netball players or athletes. This study aimed to determine the health status, medication use and attitudes towards doping of South African senior netball players.

## 2 Materials and methods

### 2.1 Study design and ethical considerations

The study was a quantitative, cross-sectional, descriptive study. Data was collected by means of an online self-report questionnaire in compliance with the Checklist for Reporting Results of Internet E-Surveys (CHERRIES) guidelines. The study received ethical approval from the Research Ethics Committee of the Faculty of Health Sciences at the University of Pretoria (REC number: 373/2022).

### 2.2 Participants (selection and description)

South African senior netball players are defined as athletes actively representing the Spar Proteas and/or athletes actively playing in professional netball competitions. The South African senior netball players studied are affiliated with Netball South Africa, which is a full member of World Netball, the sole internationally recognized governing body for netball (16, 17). A non-random, purposive sampling of a defined group of netball players was conducted. A total of 110 netball players gave written informed consent and completed the online self-report questionnaire. Following the data cleaning process, incomplete surveys were excluded, and 60 netball players’ responses were analyzed.

### 2.3 Data collection

The prevalence of chronic disease and medication used to treat chronic disease was determined. Information on medication prescribed and purchased OTC shortly before or during a competition was sampled. The validated 8-item Performance Enhancement Attitudes Scale (PEAS) was used to gather information on the netball players’ attitudes to doping (18). A pilot study was conducted on 10 senior South African netball players to assess question validity and consistency and limit survey bias. Permission was granted by the South African Netball Federation (NSA) to conduct the study. The online self-report questionnaire using the Qualtrics platform was distributed to senior netball players by NSA and the district head members via an email link provided by the researchers.

### 2.4 Statistical analysis

Descriptive statistics were used to describe the data using proportions (categorical), means (normally distributed, continuous) and medians (non-normally distributed, continuous). Doping prevalence and the accompanying 95% confidence interval were calculated.

## 3 Results

### 3.1 Participant demographics

The majority of the netball players fell within the 18–25 years age range (65%), 23% within the 25–30 years age range, and 11.67% within the 35–40 years age range.

Three netball players did not answer the question regarding netball playing experience. Just over 86% of the netball players had more than 10 years’ experience, 6.67% had 5–7 years’ experience and 1.67% had 2–4 years’ experience. A total of 90% of the netball players participated in netball more than 3 times per week, 8.33% participated 2 times per week and 1.67% participated 1 time per week.

Concerning netball playing position, 35% of the netball players played Goal Shooter, 30% played Goal Defense, 11.67% played Wing Attack, 11.67% played Goalkeeper, 6.67% played Centre, and 5% played Wing Defense.

Over 36% of the netball players played at least 2 netball games per week, 28.33% played more than 3 games per week, and 25% of the netball players played 1 game per week. Competitions played are shown in Table 1.

**Table 1:** Competitions played.

		*n* (60)	Prevalence (%)
Competitions played	University Netball (University Sports South Africa, Varsity Cup, Internal Leagues, Unspecified)	29	48.33
	Telkom Netball League	15	25.00
	Provincial Competitions (Inter Provincial Tournaments, Tshwane League, Gauteng Championships, KwaZulu-Natal super league, Ethekwini netball league)	12	20.00
	Spar national championships	9	15.00
	National Competitions	6	10.00
	Other competitions (Quad series, Suncorp Supernetball, Vitality netball super league, Mayoral games, South African championships)	5	8.33
	None right now	4	6.67
	All competitions Spar Proteas competed in	3	5.00
	Twizza	3	5.00
	District	2	3.33
	International Leagues	2	3.33
	League Unspecified	2	3.33

### 3.2 Incidence of illness

The prevalence of chronic disease was reported at 11.67% (95% CI: 4.82–22.57) utilizing the “exact method”. In particular, 1.67% of players reported being clinically diagnosed with hypertension, high cholesterol, HIV/AIDS, thyroid disease and heart disease. Furthermore, 3.33% of players reported being diagnosed with asthma, and 3.33% of players reported being diagnosed with other conditions, including depression and attention-deficit/hyperactivity disorder (ADHD).

### 3.3 Medication use

The prevalence of chronic prescription medication use was 8.33%. The most common prescription medication utilized was Symbicord turbuhaler at 3.33%. Pulmicort, Exsira, Epitec, Pritor, Amloc, Astor, Eltroxin and Concerta were reported at a prevalence of 1.67%, respectively.

The prevalence of answering YES to receiving any prescription injections, medications or utilizing any OTC medications for treating injury or illness suffered 1–6 weeks before or during competition was 66.67%. The most prevalent medication utilized was oral anti-inflammatories at 15%, followed by oral analgesics at 11.67%, platelet-rich plasma (PRP) injections at 5%, cortisone injections, cold and flu and cough syrup medication at 3.33% and anti-retroviral (ARV) drugs—Ribavirin at 1.67%.

### 3.4 Supplement use

Just over 31% of the netball players reported they were using nutritional supplements. The prevalence of nutritional supplement use was calculated with 2 missing responses. The most common nutritional supplements utilized were vitamins at 55.56%, minerals at 50%, caffeine and branched chain amino acids (BCAAs) at 33.33%, eicosapentaenoic acid (EPA), gelatin and collagen at 22.22%, creatine and herbal supplements at 11.11%, and other nutritional supplements at 22.22%. Further evaluation of the OTHER nutritional supplements revealed that anabolic steroids, fat burners and methylsulfonylmethane (MSM) were reported at 5.56% respectively.

Reasons for utilizing nutritional supplements are presented in Table 2. The most common reason was for recovery at 33.33%.

**Table 2:** Reasons for utilizing nutritional supplements.

Reason	*n* (18)	Prevalence (%)
Recovery	6	33.33
Boost immune function	4	22.22
Address Mineral and Micronutrient Deficiencies	3	16.67
Assist with training	3	16.67
Joint and tendon support	2	11.11
To maintain health	2	11.11
Reduce fatigue	1	5.56
Assist in the prevention of familial diseases	1	5.56
Relieve muscle stiffness	1	5.56
Boost energy	1	5.56

### 3.5 Prevalence of doping

The prevalence of answering NO to doping was 88.33% (95% CI: 77.43–95.18) utilizing the “exact method”. However, within the dataset seven responses were observed to be incomplete.

### 3.6 Attitudes towards doping

Attitudes towards doping are presented in Table 3. The attitudes towards doping response percentage were calculated with a mean of 9 missing responses. In general, the netball players were intolerant of doping and believed that doping is unnecessary to be competitive in netball. The netball players indicated that they believed that the pressure to perform or pressure from coaches and parents, to gain a competitive edge or improve performance, underperformance, lack of knowledge of the prescription or OTC medication, improve recovery, cope with depression, gain more skills, fear of losing a spot to younger players, to make the team, chronic disease management, and negligence of the coaching or support staff, make netball players more vulnerable to intentional or unintentional doping.

**Table 3:** Attitudes towards doping.

Performance enhancement attitude scale (PEAS) items	Mean score ± SD
Q1: Legalizing performance enhancements would be beneficial for sport.	1.79 ± 1.4
Q2: Doping is necessary to be competitive.	1.30 ± 1.0
Q3: The risks related to doping are exaggerated.	2.20 ± 1.7
Q4: Athletes should not feel guilty about breaking the rules and taking performance-enhancing drugs.	1.22 ± 0.8
Q5: Doping is an unavoidable part of competitive sport.	1.68 ± 1.3
Q6: Doping is not cheating since everyone does it.	1.31 ± 1.0
Q7: Only the quality of performance should matter, not the way athletes achieve it.	1.48 ± 1.2
Q8: There is no difference between drugs and biomechanically advantageous sport equipment that are used to optimise sports performance.	1.68 ± 1.3
PEAS total	21.20 ± 0.3

### 3.7 Attitudes and perceptions

Reasons for competing in netball are presented in Table 4. Most participants played netball to enjoy themselves and to have fun.

**Table 4:** Reasons for competing in netball.

Reason	*n* (60)	Prevalence (%)
To enjoy myself and have fun	41	68.33
To travel and gain new experiences	22	36.67
To compete to win	22	36.67
To form part of a team	17	28.33
For social interaction and being with friends	13	21.67
To relieve stress and feel better	12	20.00
Other	2	3.33
To delay the effects of aging	1	1.67
Suicide prevention	1	1.67
To be the best that I can	1	1.67

### 3.8 Anti-doping education status

Just under 26% of the netball players reported being unfamiliar with the WADA website, anti-doping rules, regulations and policies. Furthermore 24.07% of the netball players indicated that they had not heard of the WADA prohibited list. The prevalence of being familiar with the anti-doping education resources was calculated with 6 missing responses.

The netball players’ sources of anti-doping information are presented in Table 5. WADA resources and Google/Internet, accounted for the most prevalent sources consulted. The prevalence of sources of anti-doping information was calculated with 28 missing responses.

**Table 5:** Sources of anti-doping information.

Reason	Prevalence (%)
WADA resources	18.75
Google/Internet	18.75
Workshops/seminars/courses	15.63
Coaches	9.38
Qualified professional (health care provider, doping agent, member of SASCOC)	9.38
NSA	6.25
Athletes	6.25
SAIDS resources	6.25
University	6.25
At netball tournaments	3.13

NSA, Netball South Africa; SASCOC, South African Sports Confederation and Olympic Committee; WADA, World Anti-doping Agency; SAIDS, South African Institute for Drug-Free Sport.

The prevalence of answering YES to having verified with a medical care provider if the medication they prescribed is legal to use when participating in sports events was 64.81% and the prevalence of answering NO was 35.19%. Additionally, 11.11% of the surveyed netball players indicated that they have applied for a TUE for themselves or a family member competing in any sport. The prevalence of asking about the legality of medications and having ever applied for a TUE was calculated with 6 missing responses.

The prevalence of answering YES to being aware of the consequences of violating the anti-doping rules and regulations was 86.79% and the prevalence of answering NO was 13.21%. The prevalence of awareness of the consequences of anti-doping violations was calculated with 7 missing responses.

## 4 Discussion

The study aimed to determine the health status, medication use and attitudes towards doping of South African senior netball players. The main findings of the study were: (1) the prevalence of chronic disease was 11.67%. (2) The prevalence of chronic prescription medication use was 8.33%, and receiving any prescription injections, medications or utilizing any OTC medications for treating injury or illness suffered 1–6 weeks before or during competition was 66.67% and nutritional supplement use was 30%. (3) The netball players do not have a lenient attitude towards doping.

The majority of the netball players fell within the 18–25 year age range (65%), had more than 10 years of netball playing experience (86.67%) and participated in training more than 3 times per week (90%). In terms of netball positions; the most common positions played by the netball players was Goal Shooter (35%) and Goal Defence (30%). The most commonly reported competitions that the netball players competed in included university netball competitions, the Telkom Netball League, provincial competitions, and the Spar National Championships. The majority of the netball players reported playing games two times a week at a prevalence of 36.67% or three times a week at a prevalence of 28.33%.

Asthma, depression and ADHD were the most prevalent chronic diseases reported at a prevalence of 3.33%. These results concur with the literature. The research suggests that asthma is a common chronic disease reported at a prevalence of 8%–55.7% in elite athletes competing in endurance-based sport, depending on the study population and the diagnostic criteria (19, 20). However, studies conducted on elite European summer Olympic athletes reported a 16.5% prevalence of asthma across all types of sports (20). The prevalence of asthma is reported to be significantly higher in elite athletes compared to the general population due to chronic exposure to agents in athletes’ exercise environments (19). Indoor exercise environments may contain aeroallergens, including dust mites, cockroaches, animal dander and mold (21). Outdoor exercise environments may contain secondary tobacco smoke, irritant chemical fumes, traffic pollution, and high ozone levels, potentially triggering asthma (22). Additionally, coastal regions tend to be more humid whereas inland regions tend to be drier. Exposure to aeroallergens likely changes depending on how inland the region is located (23). Frequent training in these exacerbating environments leads to hyper reactivity of the respiratory mucosa and chronic inflammation and disruption of the bronchial tree (19). The data has shown that the intensity of elite level sport leads to an increased number of those with asthma, increases in bronchial hyperactivity, respiratory infections and impaired immune response (19).

A meta-analysis conducted on elite athletes competing in various sports revealed that 33.6% of elite athletes reported experiencing symptoms of anxiety/depression (21). Athletes are vulnerable to mental health disorders due to risk factors including injury, involuntary termination of an athletic career, pressure to perform, public scrutiny pressure through mainstream and social media, and limited support networks due to relocation and team group dynamics (24, 25). Additionally, the mental and physical demands imposed on elite athletes increase the likelihood of developing mental illness as the peak competitive years, and the peak age for the onset of mental illness, tend to overlap (25). However, depression is largely under-reported by athletic populations as athletes tend to perceive mental health disorders as signs of weakness (24). Additionally, data suggested that athletes lack an understanding of mental health and its influence on athletic performance (25).

Attention-deficit/hyperactivity disorder, a common brain developmental disorder, is reported in the literature at a worldwide prevalence of 2.5%–7.2% (26). The essential features of the disorder include persistent patterns of age-inappropriate inattention and/or hyperactivity/impulsivity causing dysfunction in academic, work and sport settings and interpersonal relationships since before the age of 12 (26). Attention-deficit/hyperactivity disorder is reported to be more common in elite athletes compared to the general population, since children with ADHD tend to be drawn to sport as a function of the positive reinforcement and attentional activating effects afforded by physical activity (26). In an annual report published on the number of players approved for TUEs, it was reported that 8.4% of players in Major League Baseball had approved TUEs for ADHD medication (26).

The prevalence of chronic prescription medication use was 8.33%. The most common prescription medication utilized was Symbicort turbohaler at 3.33%. Pulmicort, Exsira, Epitec, Pritor, Amloc, Astor, Eltroxin and Concerta were reported at a prevalence of 1.67%. Per the South African Institute for Drug-Free Sport's (SAIDS) medication check database, these prescription medications are permitted in and out of competition, except for Pritor and Concerta (27). Pritor tablets contain hydrochlorothiazide, a thiazide-type diuretic, and are prohibited in and out of competition (27, 28). Concerta tablets contain methylphenidate, a stimulant, and are prohibited in competition only (27).

The prevalence of receiving any prescription injections, medications or utilizing any OTC medications for the treatment of injury or illness suffered 1–6 weeks before or during competition was 66.67%. The most prevalent medication used were oral anti-inflammatories at 15%, followed by oral analgesics at 11.67%, PRP injections at 5%, cortisone injections, cold and flu and cough syrup medication at 3.33% and ARV—Ribavirin at 1.67%. Cortisone injections and certain cold, flu and cough syrup medications are prohibited in competition (27). Cortisone is classified under Section S9 glucocorticosteroids on the WADA 2024 prohibited list and is prohibited for systemic and non-systemic use in competition (27). Cold, flu and cough syrup medication that contains pseudoephedrine and ephedrine (stimulants) are prohibited in competition only (27).

Only 31.03% of surveyed netball players reported using nutritional supplements. The most common nutritional supplements utilized were vitamins, minerals, caffeine and BCAAs, EPA, gelatin and collagen, creatine, herbal and other nutritional supplements. The prevalence of nutritional supplement use in netball players is significantly less compared to other populations reported in the literature (7). It has been reported that 65% of Canadian Olympic athletes, 89% of American collegiate athletes and 87.5% of Australian athletes use nutritional supplements (7). The netball players further specified anabolic steroids, fat burners and MSM under the other nutritional supplements category.

Anabolic steroids are strictly prohibited in and out of competition by WADA (29). Fat burners and products that promote fat burning effects are more likely to contain high dosages of stimulants like caffeine, ephedrine, and methylhexaneamine (30). Caffeine has been included in the WADA 2024 Monitoring Programme, however, it is not considered a prohibited substance (29, 31). In contrast, ephedrine (urine concentrations greater than 10 micrograms per millilitre) and methylhexaneamine are prohibited substances in competition (27, 29). The report of anabolic steroid use as a nutritional supplement requires considerable synthesis. If the report is true, the netball player violates the WADA anti-doping rules and risks being suspended or sanctioned from netball if they undergo anti-doping testing. However, it cannot be ignored that the finding may be due to conformity bias wherein the netball player provided an answer they thought the researchers were looking for rather than responding truthfully. This may be further supported by the participant failing to complete the doping section of the survey. However, it is also pertinent to consider that the participant may have not wished to complete the doping section of the survey through conscious choice.

Nutritional supplements lack regulation and may unintentionally contain banned ingredients due to cross-contamination and poor hygiene practices in the production process or intentionally through purposeful inclusion without labelling (5, 32). Consequently, netball players ingesting nutritional supplements are at an increased risk for unintentional doping. The onus falls upon athletes to critically evaluate a nutritional supplement's demonstrated effectiveness and safety for ingestion in and out of competition (30). The top 3 reasons reported by the netball players as to why they utilize nutritional supplements included for recovery at 33.33%, for boosting immune function at 22.22%, and for addressing mineral and micronutrient deficiencies at 16.67%.

The study demonstrated that most netball players have a somewhat negative attitude towards doping (Total PEAS score of 21.20 ± 0.3) (18). The total 8-item PEAS score for the netball players is higher in comparison to the 8-item PEAS score reported for Korean national athletes competing in the Rio 2016 Olympic games (Total PEAS score of 13.66) (33). Total scores higher than 22 indicate that the athletes possess positive attitudes towards doping, while total scores below 21.9 denote that the athletes possess negative towards doping (18). Some of the netball players (11.76%) revealed that they slightly agreed that legalizing performance enhancements would benefit sport and that the risks related to doping are exaggerated. The study further revealed that 21.57% of netball players strongly disagreed that intentionally taking PEDs with the sole purpose of optimizing performance has long term health implications, and 29.41% of the netball players strongly disagreed that the use of chronic prescription medication assists netball players in performing as healthy individuals. These findings may suggest that the netball players are inadequately informed regarding the risks and health implications associated with doping. Additionally, the results revealed that the netball players might not understand that players taking chronic prescription medication to maintain general health does not compromise fair play. This may suggest that the current anti-doping educational guidelines and interventions may need to further emphasize these areas. However, 94.11% of the netball players disagreed that doping is necessary to be competitive, and 88.33% of the netball players revealed that they are not intentionally doping.

The top 3 reported reasons as to why the netball players think that netball players may be more vulnerable to intentional or unintentional doping include pressure to perform at 13.33%, to gain competitive advantage at 10%, and to improve performance at 6.67%. Comparative to data reported in the literature, positive attitudes towards doping include improved physical performance and energy, reductions of fear of failure through increasing the probability of winning because the playing field is perceived to be levelled, obtaining competitive advantage, modelling after sport heroes and gaining support from peers, relaxation and ability to cope with the pressure to perform well, pain reduction and rehabilitation from injury leading to sooner return to play and weight reduction (15, 18, 34).

Additional pertinent findings in this section of the survey revealed that some netball players believe that the sport is clean of doping and that the netball players have not been tested for doping since before the outbreak of COVID-19, so there are no repercussions for doping. The latter report raises concern, as this belief may influence a netball player's belief that netball is not being monitored for anti-doping violations and may possibly motivate a player with positive attitudes towards doping to commit intentional doping offences to assist them to compete to win or to gain competitive advantage, to cope with the pressures to perform and to improve performance.

The top 3 reasons reported by the netball players as to why they compete in netball include to enjoy themselves and to have fun at 68.33%, to compete to win at 36.67%, and to travel and gain new experiences at 36.67%. Goal perspective influences how individuals think, feel, and act in achievement situations like sports (35). Task and ego orientation differentiates how athletes appraise their ability, effort and performance level (35). Ego-orientated athletes value outperforming their athletic counterparts by utilizing minimal effort signifying superior competence (35). Ego-orientated athletes are more likely to adopt negative achievement behaviors, including deceptive tactics (35). Therefore, a competing to win mentality combined with positive attitudes towards doping may increase an ego-orientated netball player's likelihood of committing intentional anti-doping violations.

Regarding anti-doping education status, many netball players reported that they were familiar with the WADA website, anti-doping rules, regulations and policies and that they had heard of the WADA prohibited list. However, 25.93% of the netball players reported that they were not familiar with the WADA website, anti-doping rules, regulations and policies, and 24.07% of netball players reporting that they had not heard of the WADA prohibited list. Concerns are further raised with 13.21% of the netball players reporting that they were not aware of the consequences of committing an anti-doping violation. This supports that anti-doping education programs and interventions in netball, especially following the progression of “life as usual” following the outbreak of the COVID-19 pandemic, may require a more intensive approach.

Those netball players who reported being familiar with anti-doping rules and regulations maintained that their information sources are mostly credible. The top 3 resources included: WADA resources, internet sources, and workshops, seminars and courses. Only 6.25% of netball players use SAIDS, as an information source. This may suggest that South African athletes are unaware of SAIDS and the services and support they can offer South African athletes.

## 5 Strengths and limitations

Study limitations include convenience sampling and a relatively small sample size which requires the extrapolation of results, to the greater population of netball players, to be done with caution. However, it is important to note that all participants surveyed were affiliated with the international governing body, World Netball at the time of the study. All World Netball affiliated players are subjected to the same rules and regulations. Further, the data collected was self-reported, thus, recall bias needs to be considered. Important strengths of the study include that the study contributes to the body of knowledge on doping and netball players as there is limited research on this topic. The results can potentially be used to guide anti-doping interventions in netball players and serve as a basis for future studies.

## 6 Conclusion

The prevalence of chronic disease (11.67%) and chronic prescription medication use (8.33%) was low in the netball players however prescription and OTC medication use to treat acute injury and illness just prior or during competition was high (66.7%). Oral analgesics, oral anti-inflammatories, PRP injections, cortisone injections, cold and flu and cough syrup medication and the ARV—Ribavirin were reported to be the most prevalent medications utilized. Only 35.19% verified with a medical care provider if the medication they prescribed is legal to use when participating in sports events. The netball players do not have a lenient attitude towards doping. The majority of the netball players reported they were familiar with the WADA website, anti-doping rules, regulations and policies. However, the prescription and OTC medication use could put this cohort of netball players at risk of unintentional doping. Anti-doping education aimed at senior athletes may be beneficial to reduce the risk of unintentional doping due to medication use for injury or illness. Additionally, anti-doping testing may need to be further prioritized to screen for any anti-doping infringements in netball. Future research may include studies in netball using larger samples of participants, comparing netball players of different age groups, collecting more data from older (35–40 years) netball players, doping behaviors in netball players and the role or perspectives of athlete support personnel in netball.

## Data Availability

The datasets presented in this study can be found in online repositories. The names of the repository/repositories and accession number(s) can be found below: The datasets [GENERATED/ANALYZED] for this study can be found in the UP Research Repository [10.25403/UPresearchdata.25117865].

## References

[1] Chandler PT PS Curran JD Gabbett TJ. Physical demands of training and competition in collegiate netball players. J Strength Cond Res. (2014) 28(10):2732–7. 10.1519/JSC.0000000000000486 24983848

[2] Whitehead S WJ Cormack S Alfano H Kerss J Mooney M Weakley J. The applied sports science and medicine of netball: a systematic scoping review. Sports Med. (2021) 51(8):1715–31. 10.1007/s40279-021-01461-6 34086257 PMC8310515

[3] Van Thuyne W DF. Declared use of medication in sports. Clin J Sport Med. (2008) 18(2):143–7. 10.1097/JSM.0b013e318163f220 18332689

[4] Chad DC Thomas LS. When food becomes a drug: nonanabolic nutritional supplement use in athletes. Am J Med. (2002) 30(6):907–16. 10.1177/03635465020300062701 12435662

[5] Rosenbloom C Murray B. Risky business: dietary supplement use by athletes. Nutr Today. (2015) 50(5):240–6. 10.1097/NT.0000000000000122

[6] Van der Merwe P Grobbelaar E. Inadvertent doping through nutritional supplements is a reality. S Afr J of Sports Med. (2004) 16(2):3–7. 10.17159/2078-516X/2004/v16i2a180

[7] Dascombe BJ Karunaratna M Cartoon J Fergie B Goodman C. Nutritional supplementation habits and perceptions of elite athletes within a state-based sporting institute. J Sci Med Sport. (2010) 13(2):274–80. 10.1016/j.jsams.2009.03.005 19775936

[8] Soto-Quijano DA. The competitive senior athlete. Phys Med Rehabil Clin N Am. (2017) 28(4):767–76. 10.1016/j.pmr.2017.06.009 29031342

[9] Sekulic D Tahiraj E Zvan M Zenic N Uljevic O Lesnik B. Doping attitudes and covariates of potential doping behaviour in high-level team-sport athletes; gender specific analysis. J Sports Sci Med. (2016) 15(4):606–15. 27928206 PMC5131214

[10] Nolte K SB Krüger PE Fletcher L. Doping in sport: attitudes, beliefs and knowledge of competitive high school athletes in gauteng province. S Afr J Sports Med. (2014) 26(3):81. 10.7196/sajsm.542

[11] Ciocca M. Medication and supplement use by athletes. Clin Sports Med. (2005) 24(3):719–38. 10.1016/j.csm.2005.03.005 16004927

[12] Hamidi M Shahbazi M-A Ashrafi H. Drug Abuse in Sport: Doping. New York: Nova Science Publishers, Inc (2012). Available online at: http://site.ebrary.com/id/10726576, https://search.ebscohost.com/login.aspx?direct=true&scope=site&db=nlebk&db=nlabk&AN=602971, https://public.ebookcentral.proquest.com/choice/publicfullrecord.aspx?p=3022537

[13] Overbye M KM Pfister G. To dope or not to dope: elite athletes’ perceptions of doping deterrents and incentives. Perform Enhanc Health. (2013) 2(3):119–34. 10.1016/j.peh.2013.07.001

[14] Alaranta A Alaranta H Holmila J Palmu P Pietilä K Helenius I. Self-reported attitudes of elite athletes towards doping: differences between type of sport. Int J Sports Med. (2006) 27(10):842–6. 10.1055/s-2005-872969 16586338

[15] Judge LW Bellar D Craig B Gilreath E. The attitudes of track and field throwers toward performance enhancing drug use and drug testing. J Res. (2010) 5(2):54–61.

[16] Netball W. Africa Region. (2024). Available online at: https://netball.sport/inside-world-netball/regions-members/africa-region/ (Accessed July 8, 2024).

[17] Netball W. About Us. (2024). Available online at: https://netball.sport/inside-world-netball/about-us/ (Accessed July 8, 2024).

[18] Folkerts D Loh R Petróczi A Brueckner S. The performance enhancement attitude scale (PEAS) reached ‘adulthood’: lessons and recommendations from a systematic review and meta-analysis. Psychol Sport Exerc. (2021) 56:1–16. 10.1016/j.psychsport.2021.101999

[19] Mulic M Lazovic B Dmitrovic R Jovicic N Detanac DS Detanac DA. Asthma among elite athletes, mechanism of occurence and impact on respiratory parameters: a review of literature. Sanamed. (2020) 15(2):209–13. 10.24125/sanamed.v15i2.439

[20] Rasmussen SM Hansen ESH Backer V. Asthma in elite athletes—do they have type 2 or non-type 2 disease? A new insight on the endotypes among elite athletes. Front Allergy. (2022) 3:973004. 10.3389/falgy.2022.973004 36340019 PMC9633848

[21] Gouttebarge V Castaldelli-Maia JM Gorczynski P Hainline B Hitchcock ME Kerkhoffs GM Occurrence of mental health symptoms and disorders in current and former elite athletes: a systematic review and meta-analysis. Br J Sports Med. (2019) 53(11):700–6. 10.1136/bjsports-2019-100671 31097451 PMC6579497

[22] Chabra R Gupta M. Allergic and Environmental Induced Asthma. Treasure Island (FL): StatPearls Publishing (2018). 30252274

[23] Utell MJ Looney RJ. Environmentally induced asthma. Toxicol Lett. (1995) 82:47–53. 10.1016/0378-4274(95)03467-6 8597096

[24] Forys WJ Tokuhama-Espinosa T. The athlete’s paradox: adaptable depression. Sports (Basel). (2022) 10(7):105. 10.3390/sports10070105 35878116 PMC9320389

[25] Rice SM Purcell R De Silva S Mawren D McGorry PD Parker AG. The mental health of elite athletes: a narrative systematic review. Sports Med. (2016) 46(9):1333–53. 10.1007/s40279-016-0492-2 26896951 PMC4996886

[26] Han DH McDuff D Thompson D Hitchcock ME Reardon CL Hainline B. Attention-deficit/hyperactivity disorder in elite athletes: a narrative review. Br J Sports Med. (2019) 53(12):741–5. 10.1136/bjsports-2019-100713 31097459

[27] Sport SAIfD-F. Medication Check. (2023). Available online at: https://drugfreesport.org.za/online-medication-check/ (Accessed April 30, 2023).

[28] Medicine NLo. Hydrochlorothiazide. (2022). Available online at: https://www.ncbi.nlm.nih.gov/books/NBK430766/#:∼:text=Hydrochlorothiazide%20(HCTZ)%20is%20a%20thiazide,sodium%20chloride%20co%2Dtransporter%20system (Accessed April 30, 2023).

[29] WADA. Prohibitted List World Anti-Doping Agency. (2024). Available online at: https://www.wada-ama.org/sites/default/files/2023-09/2024list_en_final_22_september_2023.pdf (Accessed April 30, 2023).

[30] Sport SAIfD-F. Supplements and their Risks. (2023). Available online at: https://drugfreesport.org.za/supplements-and-their-risks/ (Accessed May 2, 2023).

[31] WADA. The 2024 Monitoring Program. (2024). Available online at: https://www.wada-ama.org/sites/default/files/2023-09/2024_list_monitoring_program_en_final_22_september_2023.pdf (Accessed July 8, 2024).

[32] Petróczi A Naughton DP Mazanov J Holloway A Bingham J. Limited agreement exists between rationale and practice in athletes’ supplement use for maintenance of health: a retrospective study. Nutr J. (2007) 6:34. 10.1186/1475-2891-6-34 17971239 PMC2246148

[33] Kim T Kim YH. Korean National athletes’ knowledge, practices, and attitudes of doping: a cross-sectional study. Subst Abuse Treat Prev Policy. (2017) 12(1):1–8. 10.1186/s13011-016-0086-x 28196542 PMC5309985

[34] Chan DK Hardcastle SJ Lentillon-Kaestner V Donovan RJ Dimmock JA Hagger MS. Athletes’ beliefs about and attitudes towards taking banned performance-enhancing substances: a qualitative study. Sport Exerc Perform Psychol. (2014) 3(4):241–57. 10.1037/spy0000019

[35] Stavrou NAM Psychountaki M Georgiadis E Karteroliotis K Zervas Y. Flow theory—goal orientation theory: positive experience is related to athlete’s goal orientation. Front Psychol. (2015) 6:1499. 10.1037/spy0000019 26500577 PMC4598580

